# Chromothripsis is a common mechanism driving genomic rearrangements in primary and metastatic colorectal cancer

**DOI:** 10.1186/gb-2011-12-10-r103

**Published:** 2011-10-19

**Authors:** Wigard P Kloosterman, Marlous Hoogstraat, Oscar Paling, Masoumeh Tavakoli-Yaraki, Ivo Renkens, Joost S Vermaat, Markus J van Roosmalen, Stef van Lieshout, Isaac J Nijman, Wijnand Roessingh, Ruben van 't Slot, José van de Belt, Victor Guryev, Marco Koudijs, Emile Voest, Edwin Cuppen

**Affiliations:** 1 Department of Medical Genetics, University Medical Center Utrecht, Universiteitsweg 100, Utrecht, 3584 CG, The Netherlands; 2 Department of Medical Oncology, University Medical Center Utrecht, Universiteitsweg 100, Utrecht, 3584 CG, The Netherlands; 3 Hubrecht Institute KNAW and University Medical Center Utrecht, Uppsalalaan 8, Utrecht, 3584 CT, The Netherlands

## Abstract

**Background:**

Structural rearrangements form a major class of somatic variation in cancer genomes. Local chromosome shattering, termed chromothripsis, is a mechanism proposed to be the cause of clustered chromosomal rearrangements and was recently described to occur in a small percentage of tumors. The significance of these clusters for tumor development or metastatic spread is largely unclear.

**Results:**

We used genome-wide long mate-pair sequencing and SNP array profiling to reveal that chromothripsis is a widespread phenomenon in primary colorectal cancer and metastases. We find large and small chromothripsis events in nearly every colorectal tumor sample and show that several breakpoints of chromothripsis clusters and isolated rearrangements affect cancer genes, including *NOTCH2*, *EXO1 *and *MLL3*. We complemented the structural variation studies by sequencing the coding regions of a cancer exome in all colorectal tumor samples and found somatic mutations in 24 genes, including *APC*, *KRAS*, *SMAD4 *and *PIK3CA*. A pairwise comparison of somatic variations in primary and metastatic samples indicated that many chromothripsis clusters, isolated rearrangements and point mutations are exclusively present in either the primary tumor or the metastasis and may affect cancer genes in a lesion-specific manner.

**Conclusions:**

We conclude that chromothripsis is a prevalent mechanism driving structural rearrangements in colorectal cancer and show that a complex interplay between point mutations, simple copy number changes and chromothripsis events drive colorectal tumor development and metastasis.

## Background

Colorectal cancer develops from a benign adenomatous polyp into an invasive cancer, which can metastasize to distant sites such as the liver [1]. Tumor progression is associated with a variety of genetic changes and chromosome instability often leads to loss of tumor suppressor genes, such as *APC*, *TP53 *and *SMAD4*.

High-throughput DNA sequencing has indicated that there are between 1, 000 and 10, 000 somatic mutations in the genomes of adult solid cancers [2-5]. Furthermore, next-generation sequencing has revolutionized our possibilities to profile genetic changes in cancer genomes, yielding important insights into the genes and mechanisms that contribute to cancer development and progression [5,6]. Systematic sequence analysis of coding regions in primary and metastatic tumor genomes has shown that only a few mutations are required to transform cells from an invasive colorectal tumor into cells that have the capability to metastasize [7]. Similarly, only two new mutations were identified in a brain metastasis compared to a primary breast tumor [8]. These data suggest that essential mutations needed for cancer progression occur predominantly in the primary tumor genome before initiation of metastasis [9]. In line with this hypothesis is the finding that distinct clonal cell populations in primary pancreatic carcinoma can independently seed distant metastases [10]. However, marked genetic differences between primary carcinomas and metastatic lesions do exist [11], and genotyping of rearrangement breakpoints in primary and metastatic pancreatic cancer revealed ongoing genomic evolution at metastatic sites [12].

In particular, the impact of structural genomic changes and their contribution to cancer development have recently received considerable attention [8,13-15]. Many solid tumor genomes harbor tens to hundreds of genomic rearrangements, which may drive tumor progression by disruption of tumor suppressor genes, formation of fusion proteins, constitutive activation of enzymes or amplification of oncogenes [12-17]. Rearrangements may be complex, involving multiple inter- and intra-chromosomal fusions, and often reside in regions of gene amplification [13,18,19]. Recent genome-wide copy number profiling of cancer genomes suggests that 2 to 3% of all cancers appear to contain very complex rearrangements associated with two copy number states [20,21]. These events involve complete chromosomes or chromosome arms and are proposed to result from massive chromosome shattering, termed chromothripsis [20,21]. The prevalence and impact of such complex rearrangements in heterogeneous clinical specimens of solid tumors as well as their relevance for metastasis formation are currently unclear.

Here, we describe pairwise genomic analyses of matched primary and metastatic colorectal cancer samples from four patients using genome-wide mate-pair sequencing, SNP array profiling and targeted exome sequencing to explore the genetic changes that constitute colorectal cancer formation and metastasis. We find marked differences between primary and metastatic tumors and show that chromothripsis rearrangements occur frequently in colorectal cancer samples. We conclude that chromothripsis events, along with simple point mutations and structural changes, are major contributors to somatic genetic variation in primary and metastatic colorectal cancer.

## Results and discussion

### Patterns of structural variation in primary and metastatic colorectal tumors

Paired-end sequencing has proven a powerful technique to profile genomic rearrangements in cancer genomes [13]. However, there are some limitations associated with the use of short insert paired-end libraries for detecting structural variation [22]. Long-insert paired-end sequencing (also known as long mate-pair sequencing) has the advantage of being able to detect structural changes across repetitive and duplicated sequences [19].

**Figure 1:**
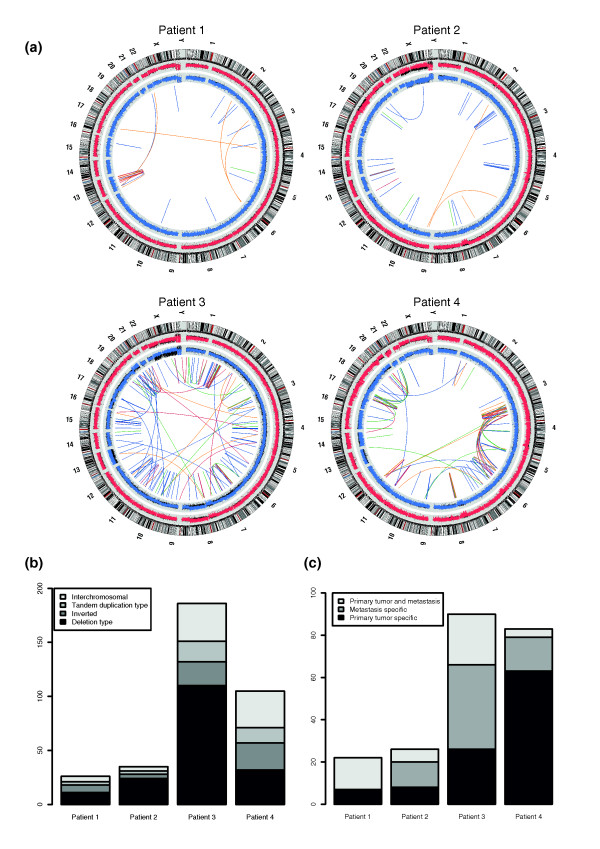
**Rearrangements in colorectal tumors detected by long mate-pair sequencing**. **(a) **Circos plots displaying rearrangements and their chromosomal locations in primary and metastatic colorectal tumor samples. Rearrangement fusion points and orientations are indicated by colored links: red, head-head; blue, tail-head; green, head-tail; orange, tail-tail (low coordinate to high coordinate). Chromosome ideograms are shown on the outer ring. The inner two rings show copy number profiles based on log R ratios derived from SNP array analysis. Red copy number plots correspond to the liver metastasis and blue plots correspond to the primary tumor. Copy number variation for matching normal colon and liver tissue are plotted in black. **(b) **Classes of rearrangements identified in tumors of the four patients. Deletion-type rearrangements have tail-head orientation, tandem duplication type rearrangements have head-tail orientation and inverted rearrangements have head-head or tail-tail orientation. **(c) **Lesion-specific presence of rearrangements in primary and metastatic tumors as based on PCR genotyping of DNA samples from primary tumor, metastasis and control tissue.

To study the landscape of structural genomic changes in fresh tumor samples, we applied genome-wide long mate-pair sequencing and complementary SNP array profiling to matching primary and metastatic colorectal cancer biopsies from four patients (Table 1; Additional file 1; Materials and methods). Parallel analysis of normal tissues allowed us to efficiently detect *de novo *somatic rearrangements in the genomes of primary and metastatic lesions. Per sample, we generated between 10 and 65 million mate-pair sequence reads with an average insert size of 2.5 to 3 kb, resulting in 10× to 48× average physical genome coverage per sample (Additional files 2 and 3). We identified 352 somatically acquired rearrangements in the four patients, including deletions (177), tandem duplications (39), inversions (58), and interchromosomal rearrangements (78) (Figure 1a, b; Additional file 4). We independently confirmed the tumor-specific presence of 222 structural changes by PCR across the rearrangement breakpoint. Intrachromosomal rearrangements were particularly prevalent in our colorectal tumor samples, similar to what has been described for other tumor types (Figure 1b) [12,14,16]. Deletion-type rearrangements formed the most common class of rearrangements, with small deletions (up to 5 kb) being more common than large deletions (Additional file 5). This is in contrast to primary breast cancer genomes, for which tandem duplications form the most common rearrangement class and deletions form the second largest class [14].

**Table 1:** Patient overview and tumor status

Patient ID	Gender	Type	Primary tumor grade	**Metastasis resection**^**a**^	**Treatment**^**b**^
Patient 1	Female	Adenocarcinoma	Moderately differentiated	3 months	No treatment
Patient 2	Male	Adenocarcinoma	Moderately differentiated	20 months	No treatment
Patient 3	Male	Adenocarcinoma	Poorly differentiated	10 months	XELOX^c ^and Bevacizumab
Patient 4	Female	Adenocarcinoma	Well differentiated	9 months	5FU, leucovorin, oxaliplatin, bevacizumab

^a^Time between primary resection and metastasis resection. ^b^Treatment after primary tumor resection. ^c^Capecitabine and oxaliplatin. 5FU, fluorouracil.

Since we sequenced both primary tumor genomes and liver metastases as well as control tissue, we could distinguish between rearrangements that were specific to both or one of these lesions. For all 222 confirmed rearrangements, we performed PCR-based breakpoint sequencing in primary tumor, metastasis and control samples (normal liver and normal colon tissue). The sensitivity of detecting a breakpoint by PCR is below 0.001% and should therefore be a reliable estimate of the presence of a rearrangement in DNA from a highly heterogeneous tumor sample [23]. Based on PCR-based breakpoint sequencing we found that, depending on the patient, between 32% and 95% of all rearrangements were specific to either the primary tumor or the metastasis (Figure 1c). There are several potential explanations for the observed differences between primary and metastatic sites: (i) changes could have occurred in the primary tumor and metastasis after dissemination to the liver; (ii) the part of the primary tumor sample that we analyzed did not contain the cells that were giving rise to the metastasis; (iii) metastatic tumor cells may have lost rearrangements that occurred in the primary tumor; and (iv) PCR may not be sensitive enough to detect breakpoints in very low numbers of cells, such as subclones in the primary tumor that may have given rise to the metastasis [10]. Given the significant overlap in somatic structural changes between primary tumors and corresponding metastases (5 to 68%; Figure 1c), we reason that many rearrangements arose in the primary tumor before metastatic spread. These overlapping rearrangements within a patient may represent early somatic rearrangements within the primary parental clone [10]. Subsequent genomic instability in the metastatic lesion may have led to additional structural changes on top of the ones that were found in the primary tumor [12]. The many primary-tumor specific rearrangements likely arose after dissemination to the liver or were present in subclones of the primary tumor that did not have the capability to metastasize. Taken together, our pairwise comparison of structural changes in colorectal tumors shows that primary and metastatic colorectal cancer genomes have rearrangements in common, but also harbor distinct patterns of structural variation.

### Chromothripsis is a common mechanism driving structural changes in primary and metastatic colorectal tumors

Mate-pair sequencing allows identification of rearrangement breakpoints at nucleotide resolution. Furthermore, mate-pair signatures involved in complex patterns of structural changes may be used to reconstruct rearranged chromosomes by linking chromosomal fragments together based on their relative orientation. We have previously used mate-pair information to resolve a complex chromothripsis event in the germline [24].

**Figure 2:**
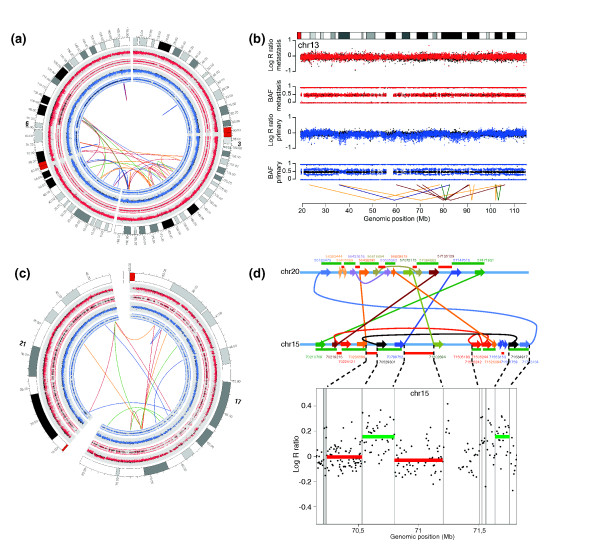
**Examples of clusters of rearrangements in primary and metastatic tumor genomes**. **(a) **A cluster of rearrangements involving chromosomes 3 and 6 specific for the primary tumor of patient 4. **(b) **A cluster of rearrangements on chromosome 13, which could be found in both the primary tumor and the liver metastasis of patient 1. **(c) **A metastasis-specific cluster of rearrangements involving chromosomes 17 and 21 of patient 4. Orientations of fusions are colored as in Figure 1. Red copy number plots and B allele frequencies correspond to the liver metastasis and blue plots correspond to the primary tumor. Copy number variation and B allele frequencies for matching normal colon and liver tissue are plotted in black. **(d) **Breakpoints and copy number changes involving a cluster of rearrangements on chromosomes 15 and 20 in the primary tumor genome of patient 3. The upper panel shows a nucleotide-resolution map of fusion points for this cluster. Lines indicate fusions between chromosomal fragments. Genomic coordinates indicate positions of breakpoints. Chromosomal fragments with both head and tail side connected to other fragments are retained, while fragments that lack any link (fusion) are supposed to be deleted. This expected pattern of retained and deleted fragments is reflected by the copy number profile for chromosome 15 (lower panel). BAF, B allele frequency.

Close examination of the landscape of genomic rearrangements in primary and metastatic samples revealed chromosomal locations where breakpoints form complex clusters (Figure 2; Additional file 6). Several mechanisms may account for the occurrence of complex rearrangements in cancer genomes [18,21,25]. Complex rearrangement patterns have been found in cancer amplicons [18], which may result from the breakage-fusion-bridge cycle following telomere dysfunction [25,26]. We do not find evidence for genomic amplification of regions involved in the complex clusters found here. Therefore, we consider it unlikely that these complex rearrangements are a result of the breakage-fusion-bridge cycle. As outlined below, we find that several complex clusters identified here resemble the chromothripsis rearrangements described recently [21].

Clusters contain short and large chromosomal fragments that have head and tail sides connected to other distant chromosomal fragments as exemplified for the cluster involving chromosomes 15 and 20 in patient 3 (Figure 2d). Furthermore, the inter- and intrachromosomal breakpoints of this cluster and most other clusters (chromosomes 17 and 21, chromosomes 3 and 6, chromosome 13) are associated with copy number changes (Additional file 7), leading to two copy number states: high for retained fragments (that is, with head and tail sides connected to other chromosomal fragments) and low for lost fragments (no connection to other fragments) (Figure 2d). Such alternating high and low copy number states are a striking feature of chromothripsis clusters identified previously [21]. However, the copy number changes we observed were not always as pronounced as previously reported [21]. This may be due to the fact that we studied heterogeneous tumor biopsies in our study as compared to clonally derived homogeneous cell lines in the previous study.

For the clusters on chromosome 1 in patient 3, chromosomes 3 and 6 in patient 4 and chromosomes 17 and 21 in patient 4, we observed that cluster boundaries extend to telomeric regions (Additional file 8), representing another characteristic that has been described as a hallmark of chromothripsis [21].

Based on sensitive PCR genotyping of breakpoints, several chromothripsis clusters displayed exclusive presence in either the primary tumor or the metastasis (Figure 2; Additional files 4, 9 and 10), further supporting the notion that they occurred as single simultaneous events since a progressive model would more likely have resulted in the presence of at least some of the breakpoints in the corresponding lesion.

Capillary sequencing of PCR fragments across breakpoints allowed us to determine sequence characteristics of breakpoint regions. We characterized 159 fusion points at nucleotide resolution (Additional file 11), of which 69 fall within complex chromothripsis clusters. There were no major differences in breakpoint characteristics for rearrangements within or outside complex clusters. Overall, we found that 38% were blunt-ended fusions and another 40% contained several nucleotides of microhomology, the majority of the fusion points having microhomology of 1 to 3 bp. For 22% of fused segments we observed insertions of short nucleotide stretches, mostly below 6 bp, which likely represent non-templated nucleotides, which are often seen for double-strand breaks repaired by non-homologous end-joining [27,28]. Next, we determined the overlap of breakpoints with repeat annotation (long interspersed nuclear elements (LINEs), short interspersed nuclear elements (SINEs), long terminal repeat (LTR) retrotransposons, DNA repeats). However, we could not identify significant association of somatic breakpoints with any of these repeat classes when compared to a set of randomly sampled positions across the genome (Fisher exact, *P *= 0.5). The sequence characteristics of fusion points that we observed here resemble those that have been detected in various other cancers [12,14,15,19], and are in line with a process of non-homologous end-joining-mediated repair of double-strand DNA breaks [21,27,28].

Overall, we conclude that small and large chromothripsis events result from massive double-strand breaks and are frequently occurring in primary and metastatic colorectal cancer.

### Chromothripsis clusters contribute to tumorigenesis in conjunction with point mutations, copy number changes and structural rearrangements

**Figure 3:**
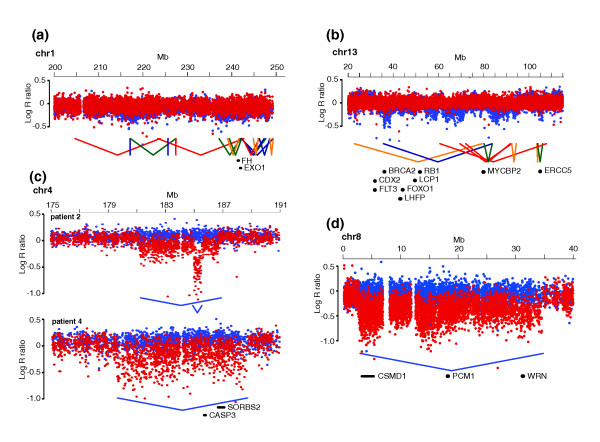
**Cancer-related genes affected by rearrangements breakpoints**. **(a) **Disruption of *EXO1 *and *FH *(fumarate hydratase) by rearrangement breakpoints in a metastasis-specific cluster on chromosome 1 in patient 3. **(b) **Disruption of *MYCBP2 *by a rearrangement breakpoint in a cluster on chromosome 13 in patient 1. Genes from the Cancer Genome Census are also depicted for this cluster. **(c) **Disruption of *SORBS2 *by metastasis-specific deletions in patients 2 and 4. **(d) **Disruption of *CSMD1 *by a metastasis-specific deletion in patient 2.

Recent studies have shown that complex rearrangements may promote cancer progression through disruption of tumor suppressor genes, or generation of fusion genes [14,15,19,21]. In addition, cancer amplicons frequently center on oncogenes, such as *ERBB2 *and *MYC *[18]. To understand the contribution of chromothripsis clusters to tumor growth and metastasis, we analyzed the breakpoint regions for the presence of cancer genes. One breakpoint of the cluster on chromosome 1 in patient 3 disrupts the fumarate hydratase gene (*FH*), which is a tumor suppressor frequently mutated in renal cell cancer (Figure 3a) [29]. Another rearrangement in the same cluster disrupts *EXO1*, which has tumor suppressor activity and may act together with *APC *to promote gastrointestinal tumor formation [30]. In patient 1, we identified a cluster on chromosome 13, and one of the breakpoints disrupts *MYCBP2 *(Figure 3b). In addition, there are several cancer related genes from the Cancer Gene Census within the boundaries of this cluster and these may be affected by one of the numerous rearrangements in this cluster [31]. Besides complex clusters, we identified a range of isolated structural rearrangements for which breakpoints affect cancer genes, such as *NOTCH2*, *FHIT*, *MLL3 *and *ETV6 *(Additional file 4) [31]. We also detected several genes that form hotspots of rearrangements in several patients (Additional file 12). For example, *PARK2 *is a tumor suppressor gene known to contain frequent deletions in colorectal cancers [32]. We identified several independent deletions of *PARK2 *in primary and metastatic tumors of patients 3 and 4. Although *PARK2 *lies in a common fragile site, which explains the frequent deletions in this gene, it may function as a tumor suppressor and disruption of *Park2 *increases adenoma development in *Apc *mutant mice [32,33]. Interestingly, patient 4 carries two independent *APC *point mutations in the primary tumor and the metastasis, respectively (see below; Table 2). We also identified several independent rearrangements in *FHIT*, *WWOX, PRKG1 *and *MACROD2 *in multiple patients. All of these genes are located at common fragile sites and have been found to contain rearrangements in several cancers [12,34].

**Table 2:** Point mutations identified in the cancer mini-exome of patients 1 to 4

	Patient 1		Patient 2		Patient 3		Patient 4	
	
Gene	TC	LM	TC	LM	TC	LM	TC	LM
*APC*	5:112175523 T/-	5:112175523 T/-	E1536* (5:1121758970)	E1536* (5:1121758970)	Y1376* (5:112175419)	Y1376* (5:112175419)	5:112128152 C/-	R499* (5:112162891)
			R876* (5:112173917)	R876* (5:112173917)				
*DDR2*				H340D (1:162731163)				
*KRAS*			G12A (12:25398284)	G12A (12:25398284)				G12A (12:25398284)
*PTPRF*			D562G (1:44058144)	D562G (1:44058144)				
*SMAD2*				R321* (18:45374882)				
*SMAD4*				L495P (18:48604662)				
*TP53*					R273C (17:7577121)	R273C (17:7577121)	R175H (17:7578406)	C275W (17:7577113)
*MLL3*	I155T (7:152012349)	I155T (7:152012349)						
*PARP14*	Q1332P (3:122437236)	Q1332P (3:122437236)						
*PIK3CA*	E545K (3: 178936091)	E545K (3: 178936091)					E545K (3: 178936091)	
*KDR*			R1032* (4:55956221)					
*PRKCD*				T419I (3:53220352)				
*RFC1*			4:39290432 T/C					
*EXOC4*	K765R (7:133682332)							
*TSC1*					R288C (9:135787720)			
*FGFR2*	R399* (10:123274723)	R399* (10:123274723)						
*NUP98*					H1647D (11:3704460)	H1647D (11:3704460)		
*ERBB3*			V104M (12:56478854)	V104M (12:56478854)				
*RASA3*				V117M (13:114806499)				
*DNAH9*			R4106H (17:11837216)					
*TAOK1*	K484M (17:27835026)	K484M (17:27835026)						
*ATRX*							X:76845412 +A	
*TTN*	K13350N (2:179445230)			H8533Y (2:179571272)				
				E4246K (2:179604510)				
*EPHA4*				2:222298957 +T				

TC, colon tumor; LM, liver metastasis. Genomic coordinates are based on the hg19 genome build.

To gain insight into the contribution of point mutations to tumor development in these and other cancer-relevant genes in our tumor samples, we performed next-generation sequencing-based mutational profiling of a cancer mini-exome in all 16 tumor and control samples (1, 296 genes; Materials and methods). We found canonical disrupting mutations in *APC*, *TP53*, *SMAD2 *and *SMAD4 *as well as *KRAS *(*G12A*) activation in several patients (Table 2) [1]. For patient 2 we identified the same mutations in *KRAS*, *APC *and *PTPRF *in both primary and metastatic tumors. However, mutations in *SMAD2 *and *SMAD4 *could only be detected in DNA from the metastatic tissue. In contrast, the tumor genomes of patient 4 contained mutations in *APC*, *KRAS *and *TP53*, but both primary tumor and metastasis carried their own private mutations in these genes. These data complement the mate-pair and copy number data, which also show overlapping mutations but also many distinct genetic variations in primary and metastatic samples, which may affect cancer genes in a lesion-specific manner (Figure 1c). For example, we identified metastasis-specific recurrent deletions of *CASP3 *and *SORBS2 *or deletion of *CSMD1 *(Figure 3c, d) [35,36]. Interestingly, *SORBS2*, which is also known as *ArgBP2*, is repressed during oncogenic transformation of the pancreas and the protein was implicated in cell adhesion and migration [36]. Furthermore, *CSMD1 *mutations have been found particularly in advanced colorectal tumors, suggesting a role in metastasis formation [35]. Therefore, the distinct genetic changes in metastastic samples compared to corresponding primary tumors likely contribute to metastasis formation or provide advantage to tumor growth at metastatic sites (liver).

These data emphasize that comprehensive genetic analysis at the nucleotide as well as structural level of both primary tumor and metastasis is needed to outline an effective targeted treatment strategy for colorectal cancer.

## Conclusions

Our data show that clusters of complex genomic rearrangements occur frequently in primary and metastatic colorectal tumors. Based on the features of these complex rearrangement clusters, we find that chromothripsis is a common driver of genetic changes in colorectal cancer. We conclude that complex chromothripsis events in conjunction with simple copy number changes and point mutations shape the dynamic architecture of colorectal cancer genomes and all together provide the genetic basis for tumor growth and metastasis. Therefore, the impact of chromothripsis on tumor development and evolution may be greater than previously anticipated [21].

The molecular mechanisms that drive chromothripsis are unclear, but the characteristics of break points suggest that chromosome shattering occurred randomly, yet regionally, as a result of double-strand breaks and that chromosomal fragments are likely repaired by non-homologous end-joining [21,24]. If the reshuffling of genetic information poses any benefit to the cell, chromothripsis clusters may drive tumor formation and metastases. A complex cluster could also be a passive genetic event - for example, when coinciding with a growth promoting mutation in the same cell. While the observation that some complex clusters are uniquely present in primary or metastatic lesions could be supportive of this hypothesis, it could also be that chromothripsis events provide a selective advantage specific for the molecular environment of either the primary tumor or the metastasis.

The distinct genetic mutation patterns in primary and metastatic tumors, illustrate the need for much more comprehensive screening of cancer genomes than is currently common practice, including profiling of (complex) structural changes along with coding mutations in primary and metastatic lesions.

## Materials and methods

### Samples

The research in this study conformed to the Declaration of Helsinki of the World Medical Association concerning human material/data and experimentation. The Medical Ethics Committee (METC) of the University Medical Centre Utrecht, The Netherlands approved the genetic analysis of DNA from tumor and normal tissues of the patients described in this paper. Tissue samples were previously acquired as part of a series of routine diagnostic and pathological analyses in our hospital.

We performed mate-pair sequencing on DNA from tumor biopsies and control samples from four patients with colorectal adenocarcinoma attending University Medical Center Utrecht, The Netherlands. For each patient, we obtained DNA from the primary colon tumor, normal colon tissue, liver metastasis and normal liver tissue. We assessed tumor content of biopsies by microscopic analysis of stained cryosections (tumor content > 80%).

### Preparation of mate-pair libraries and SOLiD sequencing

Mate-paired libraries were generated from 50 to 100 μg DNA isolated from tumor and control samples. Mate-pair library preparation was essentially as described in the SOLiDv3.5 library preparation manual (Applied Biosystems, Carlsbad, California, USA). We performed two genomic DNA size selections per library: one after shearing and one after CAP adaptor ligation. Libraries were cloned and 384 clones per library were picked for capillary sequencing to assess the presence of adaptors, insert sizes and chimeric molecules. Chimeric molecules were identified based on a tag distance > 100 kb. On average, we observed between 5% and 15% present chimeric molecules per library. We sequenced 2× 50-bp mates for each library on one or two quadrants of a SOLiD V4 sequencing slide. Mate-pair sequencing data are available from the Sequence Read Archive of the European Nucleotide Archive (ENA SRA) under accession number [SRA:ERP000875].

### Bioinformatic analysis of mate-pair reads

The F3 and R3 mate-pair tags were mapped independently to the human reference genome (GRCh37/hg19) using BWA software V0.5.0 with the following settings: -c -l 25 -k 2 -n 1 [37]. Mate-pair tags with unambiguous mapping were combined and split into local (< 100 kb) and remote (> 100 kb) mate-pair sets. Local mate-pairs were further split into mate-pairs with normal orientation of the tags relative to each other, mate-pairs with inverted tags and mate-pairs with everted tags [24].

Deletions were called from local mate-pairs with correct orientation and with a mate-pair span in the top 0.5% percentile of the mate-pair size distribution. Tandem duplications were called from local mate-pairs with everted orientation and inversions were called from local mate-pairs with inverted orientation. Mate-pairs were clustered based on overlapping mate-pairs with a maximal tag distance of two times the average library insert size. The remote (inter-chromosomal and intra-chromosomal > 100 kb) mate-pairs were clustered independently of the relative orientation of the mate-pair tags. The orientation of the different mate-pair tags in a cluster relative to each other is indicated by H (or h for the minus strand) when the tag has its 'head' side (the side that points towards the start of the chromosome) opposed to the pairing tag and T (or t for the minus strand) when a tag has its 'tail' side (the side that points towards the end of the chromosome) opposed to the pairing tag. Mate-pair clustering was performed per patient (four samples) and tumor-specific rearrangements were selected based on clusters without overlapping mate-pairs derived from normal tissue samples. Tumor-specific rearrangements were confirmed by PCR across the breakpoint in primary tumor, metastasis and normal liver and colon samples. Rearrangement fusion points were visualized by Circos software [38].

### SNP array analysis

DNA from all 16 tumor and control samples was analyzed by Illumina Cyto12 SNP arrays according to standard procedures (Illumina, San Diego, California, USA). Copy number changes and allelic profiles were derived from log R ratios and B allele frequencies that are provided by the Illumina Genomestudio package. Since overall copy number changes in the heterogeneous samples that we analyzed are not as marked as in clonally derived cell lines, we used custom scripts to detect areas with low or high log R ratio values (increase in copy number is defined as: a positive shift (> 0.1) in average log R ratio compared to a control sample (healthy colon or liver tissue from the same patient), and a decrease in copy number is defined as a negative shift (> 0.1) in log R ratio compared to the control sample. For both positive and negative changes, we required at least 12 consecutive deviating probes, while allowing a maximum of 2 probes that do not meet the criterion. Copy number changes were further substantiated by changes in average B allele frequency for heterozygous positions relative to control samples (average B allele frequency shift > 0.05, also found in a minimum of 12 sequential probes, including a 2 probe 'mismatch' cutoff). The resulting copy variable regions were manually curated based on B allele frequency plots and log R ratio plots of tumors and matching healthy samples. SNP array data were submitted to the National Center for Biotechnology Information Gene Expression Omnibus archive and are available under accession number [GEO:GSE32711].

### Mutational profiling

Mutational analysis of 1, 296 kinases and cancer-related genes was performed by multiplexed enrichment of barcoded fragment libraries from all 16 samples [39]. Capturing was done using a custom-designed Agilent 244K array with 60-mer tiled probes on both strands [40]. The pool of enriched libraries was sequenced on one slide of a SOLiD3.5 instrument. Data were mapped to the reference genome (GRCh37/hg19) using BWA (-c -l 25 -k 2 -n 1). SNP calling was done using a custom analysis pipeline that identifies mutations with a non-reference allele frequency larger than 15% and a coverage of at least 10×. Sequencing data are available from the Sequence Read Archive of the European Nucleotide Archive (ENA SRA) under accession number [SRA:ERP000875]. All identified variants were validated by PCR and capillary sequencing.

## Abbreviations

bp: base pair; PCR: polymerase chain reaction; SNP: single-nucleotide polymorphism.

## Competing interests

The authors declare that they have no competing interests.

## Authors' contributions

WK conceived and designed the study and performed the experiments and bioinformatic analysis and wrote the paper. MH performed bioinformatic analysis of array data. OP performed the breakpoint sequencing and analyzed the data. MT generated mate-pair libraries. IR performed SOLiD sequencing and generated fragment libraries. JV designed the study and contributed patient material. MR performed analysis of mate-pair sequencing data. SL performed analysis of targeted-exome sequencing data. IN performed analysis of targeted-exome sequencing data and designed the capture array. WR performed breakpoint sequencing. RS performed SNP array analysis. JB generated mate-pair libraries. VG performed analysis of mate-pair sequencing data. MK analyzed breakpoint regions and supervised experiments. EV conceived and supervised the study and wrote the paper. EC conceived, designed and supervised the study and wrote the paper.

## Supplementary Material

Additional file 1 **A flow-diagram of the procedure for detecting tumor-specific rearrangements**. Click here for file

Additional file 2 **Mean insert sizes of mate-pair libraries**. Click here for file

Additional file 3 **Table with SOLiD sequencing statistics of mate-pair libraries from tumor samples and healthy tissues**. Click here for file

Additional file 4 **Table with all tumor-specific structural rearrangements identified by mate-pair sequencing in the four patients**. Click here for file

Additional file 5 **Size distribution of tumor-specific deletions in four patients**. Click here for file

Additional file 6 **Three examples of clusters of rearrangements in colorectal tumor genomes**. Click here for file

Additional file 7 **Copy number changes coinciding with breakpoints of rearrangement clusters**. Click here for file

Additional file 8 **Log R ratios and B allele frequencies for chromosomes affected by chromothripsis**. Click here for file

Additional file 9 **PCR gel of genomic rearrangements within clusters on chromosomes 17 and 21 and chromosomes 3 and 6**. Click here for file

Additional file 10 **Table indicating the presence of complex rearrangement clusters in primary and metastatic tumors**. Click here for file

Additional file 11 **Sequence characteristics of tumor-specific fusion points**. Click here for file

Additional file 12 **Hotspots of rearrangements in *PARK2 *and *MACROD2***. Click here for file
