# Supplementary material for: Chromothripsis is a common mechanism driving genomic rearrangements in primary and metastatic colorectal cancer
Source: Genome Biol. 2011 Oct 19;12(10):R103. doi: 10.1186/gb-2011-12-10-r103 (PMC3333773; doi:10.1186/gb-2011-12-10-r103)
Supplement: Additional file 1 — A flow-diagram of the procedure for detecting tumor-specific rearrangements. [file gb-2011-12-10-r103-S1.PDF]

Additional data file 1

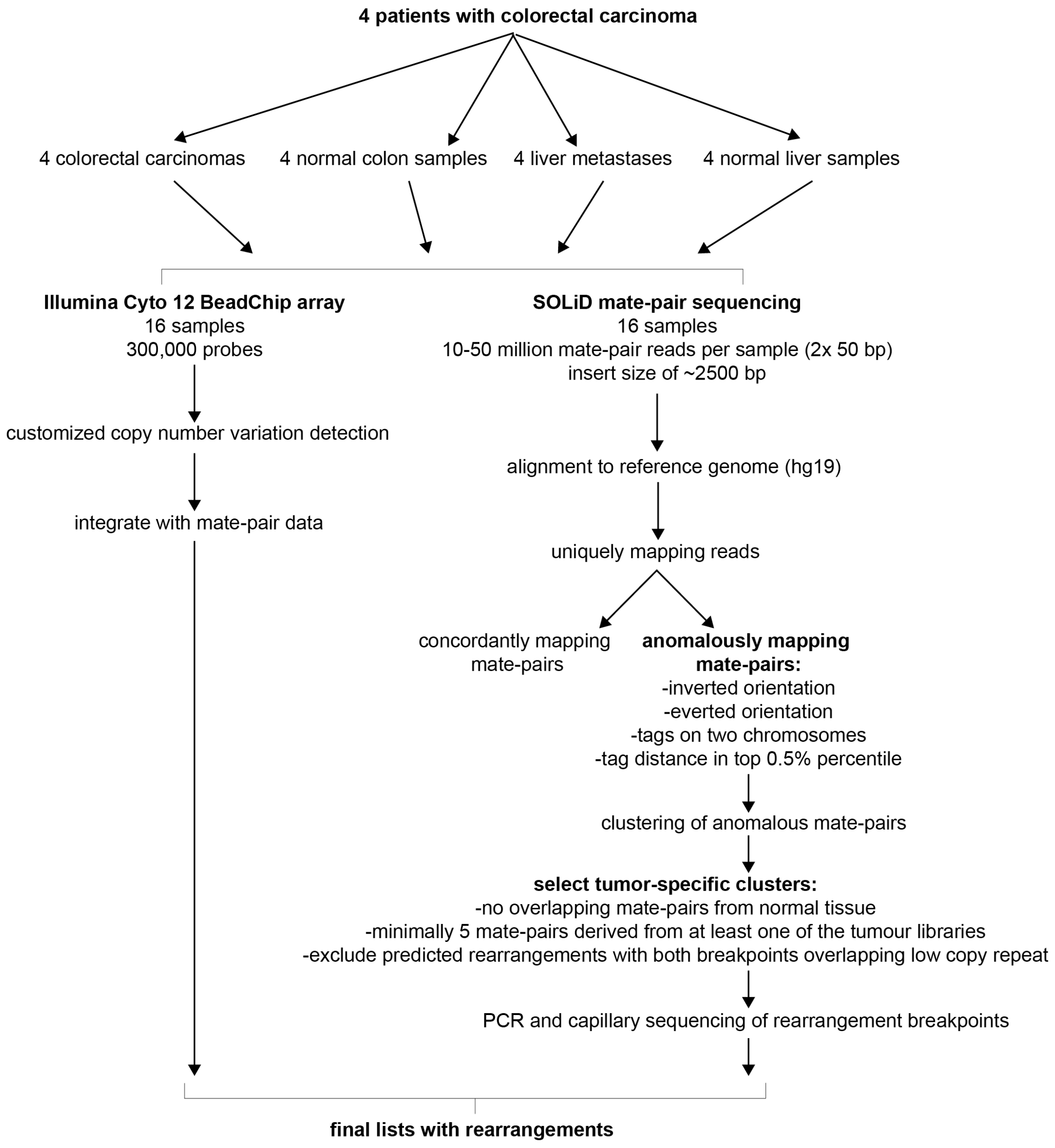

Flow diagram of the procedure for detecting tumor specific rearrangements and copy number changes.
