# Supplementary material for: Chromothripsis is a common mechanism driving genomic rearrangements in primary and metastatic colorectal cancer
Source: Genome Biol. 2011 Oct 19;12(10):R103. doi: 10.1186/gb-2011-12-10-r103 (PMC3333773; doi:10.1186/gb-2011-12-10-r103)
Supplement: Additional file 2 — Mean insert sizes of mate-pair libraries. [file gb-2011-12-10-r103-S2.PDF]

Additional data file 2

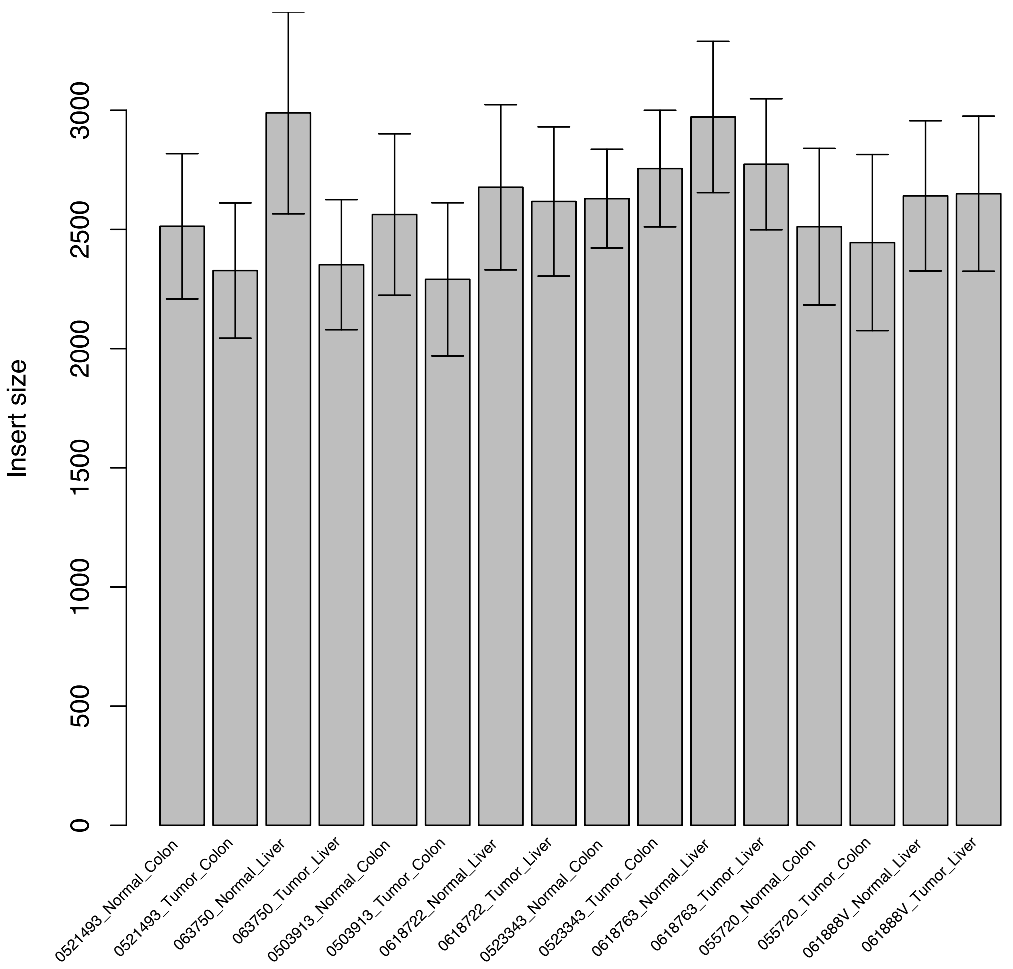

Mean insert sizes of mate-pair libraries. The mean insert sizes were calculated based on the local mate-pair clones for each library, i.e. the mate-pair clones with a tag distance smaller than 100 kb.
