# Supplementary material for: Chromothripsis is a common mechanism driving genomic rearrangements in primary and metastatic colorectal cancer
Source: Genome Biol. 2011 Oct 19;12(10):R103. doi: 10.1186/gb-2011-12-10-r103 (PMC3333773; doi:10.1186/gb-2011-12-10-r103)
Supplement: Additional file 3 — Table with SOLiD sequencing statistics of mate-pair libraries from tumor samples and healthy tissues. [file gb-2011-12-10-r103-S3.PDF]

Additional data file 3

SOLiD sequencing statistics of mate-pair libraries from tumor samples and healthy tissues

			reads					pairs						
**sample name**	**F3 reads**	**R3 reads**	**mapped F3 reads**	**mapped R3 reads**	**uniquely mapped F3**	**uniquely mapped R3**	**upper 0.5%[a,d]**	**local[a,e]**	**lower 0.5%[a,d]**	**inverted[a]**	**everted[a]**	**remote[b]**	**normal[a]**	**anomalous[c]**
patient 1 Normal Colon	64821407	64821407	44056257	52171892	40220399	48076247	178860	31810021	133860	4471	9781	1684600	31497301	2011572
patient 1 Tumor Colon	70126662	70126662	50314321	55978489	45918786	51481670	169759	31963612	134053	13111	11110	4906814	31659800	5234847
patient 1 Normal Liver	79789173	79789173	25898112	53720089	22452241	48431652	49424	8837879	43522	3145221	525905	2665707	8744933	6429779
patient 1 Tumor Liver	92773388	92773388	31487067	65017626	27782105	59290029	75116	13611256	59458	5707	3029	6308685	13476682	6451995
patient 2 Normal Colon	116090682	116092264	82718626	86295379	75575604	79111379	305980	55059891	234140	9134	17800	3676129	54519771	4243183
patient 2 Tumor Colon	116361544	116361740	81558705	85610186	74834613	78758565	284722	54860193	209263	9566	14617	3939320	54366208	4457488
patient 2 Normal Liver	105038401	105038408	74632113	78842502	68221282	72222347	269805	49021783	207985	8799	15677	5711503	48543993	6213769
patient 2 Tumor Liver	107299061	107299061	76531185	77715945	70470184	71736827	282830	51674172	225908	8891	11790	3270549	51165434	3799968
patient 3 Normal Colon	64493007	64493007	38342973	49248242	33953160	44379098	118799	22279574	104839	4796	9003	3705639	22055936	3943076
patient 3 Tumor Colon	62850908	62850908	36467462	47059308	32691844	42808265	120757	22342279	90887	5850	5267	2327339	22130635	2550100
patient 3 Normal Liver	103581735	104186075	64417151	16919730	58249355	15271056	48162	9201553	40151	1628	2083	699633	9113240	791657
patient 3 Tumor Liver	311225929	311684614	156602737	135823779	142806240	124248507	344006	58482351	245473	9092	13502	3537512	57892872	4149585
patient 4 Normal Colon	174288124	174288127	91133677	125568315	83748034	115782528	325735	62691683	283403	10122	23392	3247259	62082545	3889911
patient 4 Tumor Colon	175513797	175513953	74398343	109939900	68284177	101009636	234464	45389774	205959	10260	14944	4691246	44949351	5156873
patient 4 Normal Liver	91151259	91151259	32123263	63187541	28562184	57613264	107225	19565566	84306	6169	12946	1532273	19374035	1742919
patient 4 Tumor Liver	91093917	91093917	35112193	63112392	31352055	57810540	105051	20205544	80070	5404	10189	3147052	20020423	3347766

[a] *pairs with tag distance smaller than 100kb*

[b] *pairs with tag distance larger than 100kb*

[c] *total anomalous pairs*

[d] *pairs with tag distance in upper or lower 0.5% interval of local pairs*

[e] *correctly oriented pairs*
