# Supplementary material for: Chromothripsis is a common mechanism driving genomic rearrangements in primary and metastatic colorectal cancer
Source: Genome Biol. 2011 Oct 19;12(10):R103. doi: 10.1186/gb-2011-12-10-r103 (PMC3333773; doi:10.1186/gb-2011-12-10-r103)
Supplement: Additional file 5 — Size distribution of tumor-specific deletions in four patients. [file gb-2011-12-10-r103-S5.PDF]

Additional data file 5

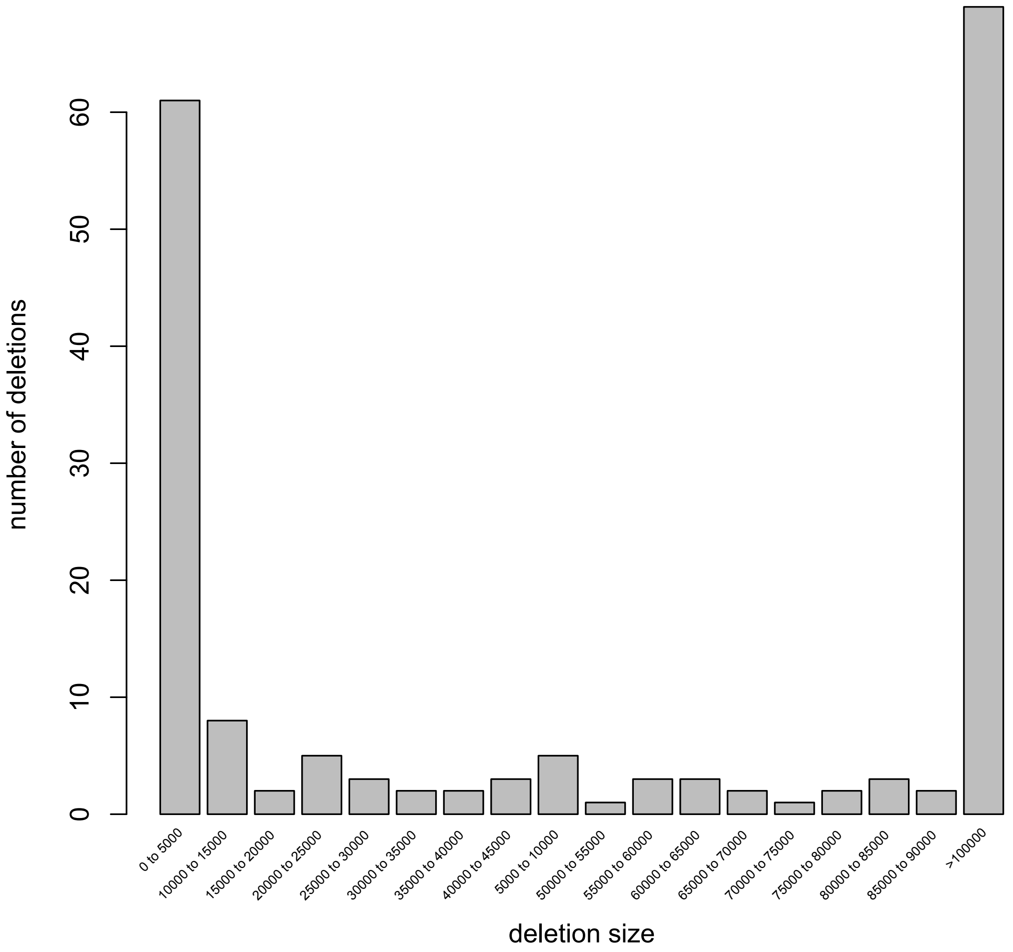

Size distribution of tumor-specific deletion-type rearrangements in four patients. Deletion sizes are estimated from the mate-pair clusters using the inner coordinates (e1 and s2) from Additional data file 4. Deletions smaller than 5kb occur most frequently. Note that some apparent deletion-type rearrangements could be part of a large rearrangement cluster and therefore may not be true deletions, but represent fusions of remote chromosomal segments.
