# Supplementary material for: Chromothripsis is a common mechanism driving genomic rearrangements in primary and metastatic colorectal cancer
Source: Genome Biol. 2011 Oct 19;12(10):R103. doi: 10.1186/gb-2011-12-10-r103 (PMC3333773; doi:10.1186/gb-2011-12-10-r103)
Supplement: Additional file 6 — Three examples of clusters of rearrangements in colorectal tumor genomes. [file gb-2011-12-10-r103-S6.PDF]

Additional data file 6

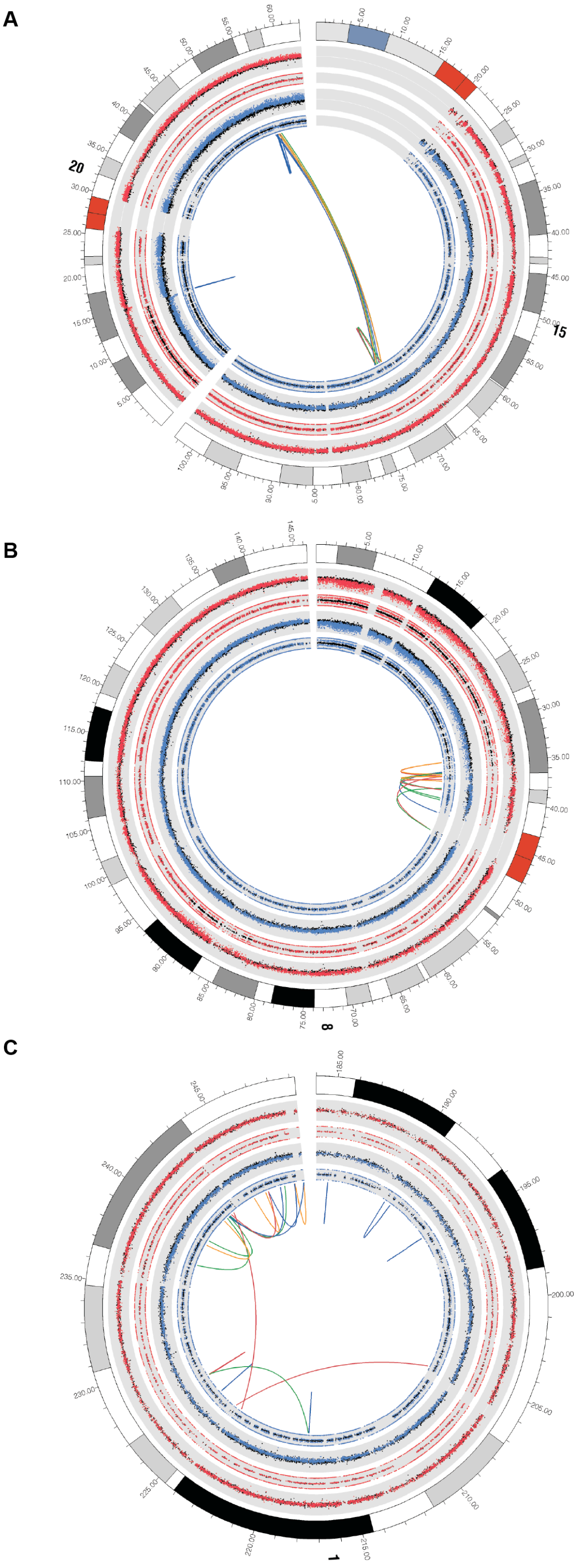

Examples of clusters of rearrangements in colorectal tumor genomes. (A) Cluster between chromosomes 15 and 20 in the primary tumor genome of patient 3. (B) Cluster on chromosome 8 in the primary tumor genome and the metastasis of patient 4. (C) Cluster on chromosome 1 in the metastasis of patient 3. Log R ratios and B allele frequencies of SNP-array data are plotted in the inner circles. Red plots correspond to data derived from

metastatic samples and blue plots correspond to data derived from the primary tumors. Black data points are derived from matching control samples.
