# Supplementary material for: Chromothripsis is a common mechanism driving genomic rearrangements in primary and metastatic colorectal cancer
Source: Genome Biol. 2011 Oct 19;12(10):R103. doi: 10.1186/gb-2011-12-10-r103 (PMC3333773; doi:10.1186/gb-2011-12-10-r103)
Supplement: Additional file 7 — Copy number changes coinciding with breakpoints of rearrangement clusters. [file gb-2011-12-10-r103-S7.PDF]

Additional data file 7

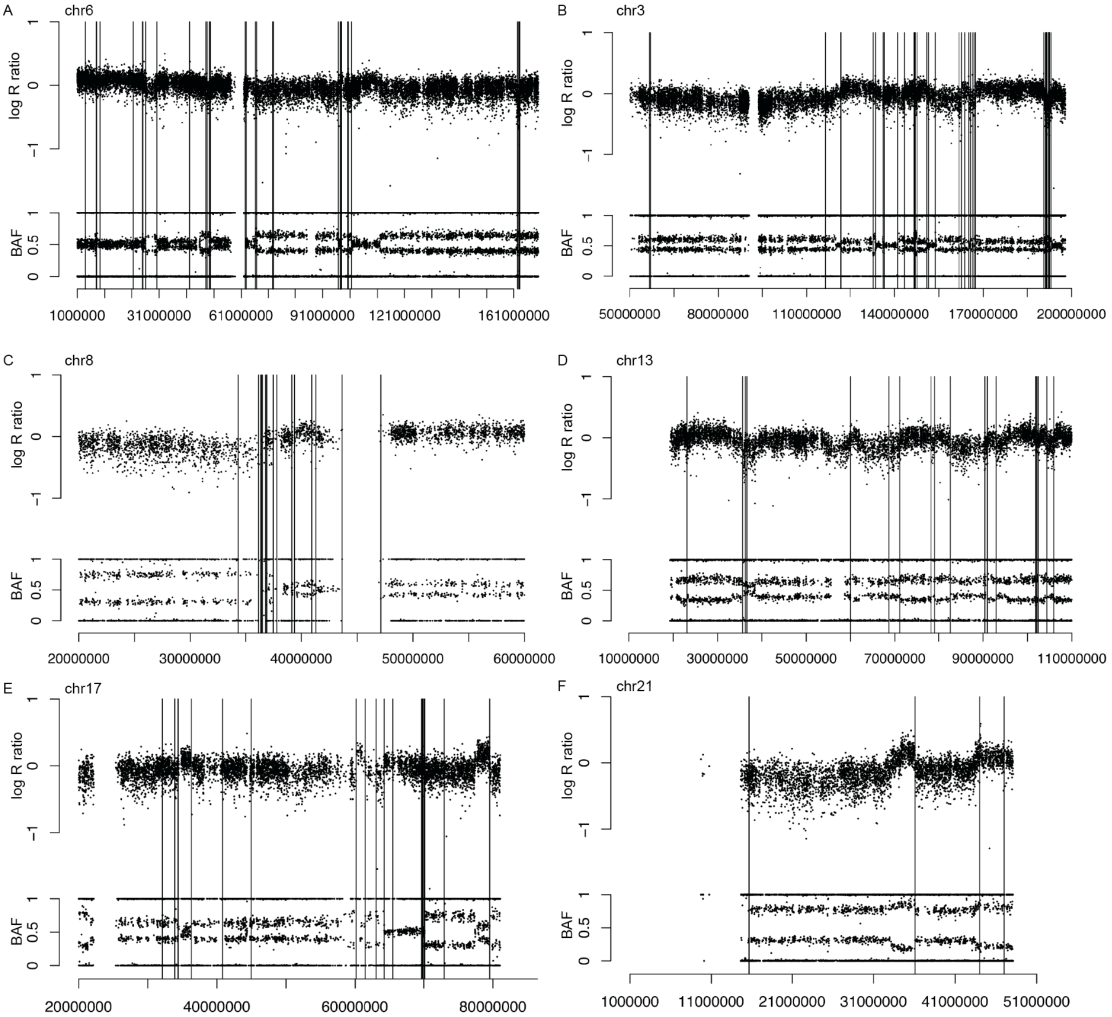

Copy number changes coinciding with breakpoints of rearrangement clusters. Copy number changes are depicted for clusters on chr 3-6 (A, B), chr 8 (C), chr 13 (D) and chr 17-21 (E, F). Breakpoints are represented by vertical lines. Copy number changes are plotted as log R ratios. BAF, B allele frequencies.
