# Supplementary material for: Chromothripsis is a common mechanism driving genomic rearrangements in primary and metastatic colorectal cancer
Source: Genome Biol. 2011 Oct 19;12(10):R103. doi: 10.1186/gb-2011-12-10-r103 (PMC3333773; doi:10.1186/gb-2011-12-10-r103)
Supplement: Additional file 8 — Log R ratios and B allele frequencies for chromosomes affected by chromothripsis. [file gb-2011-12-10-r103-S8.PDF]

Additional data file 8

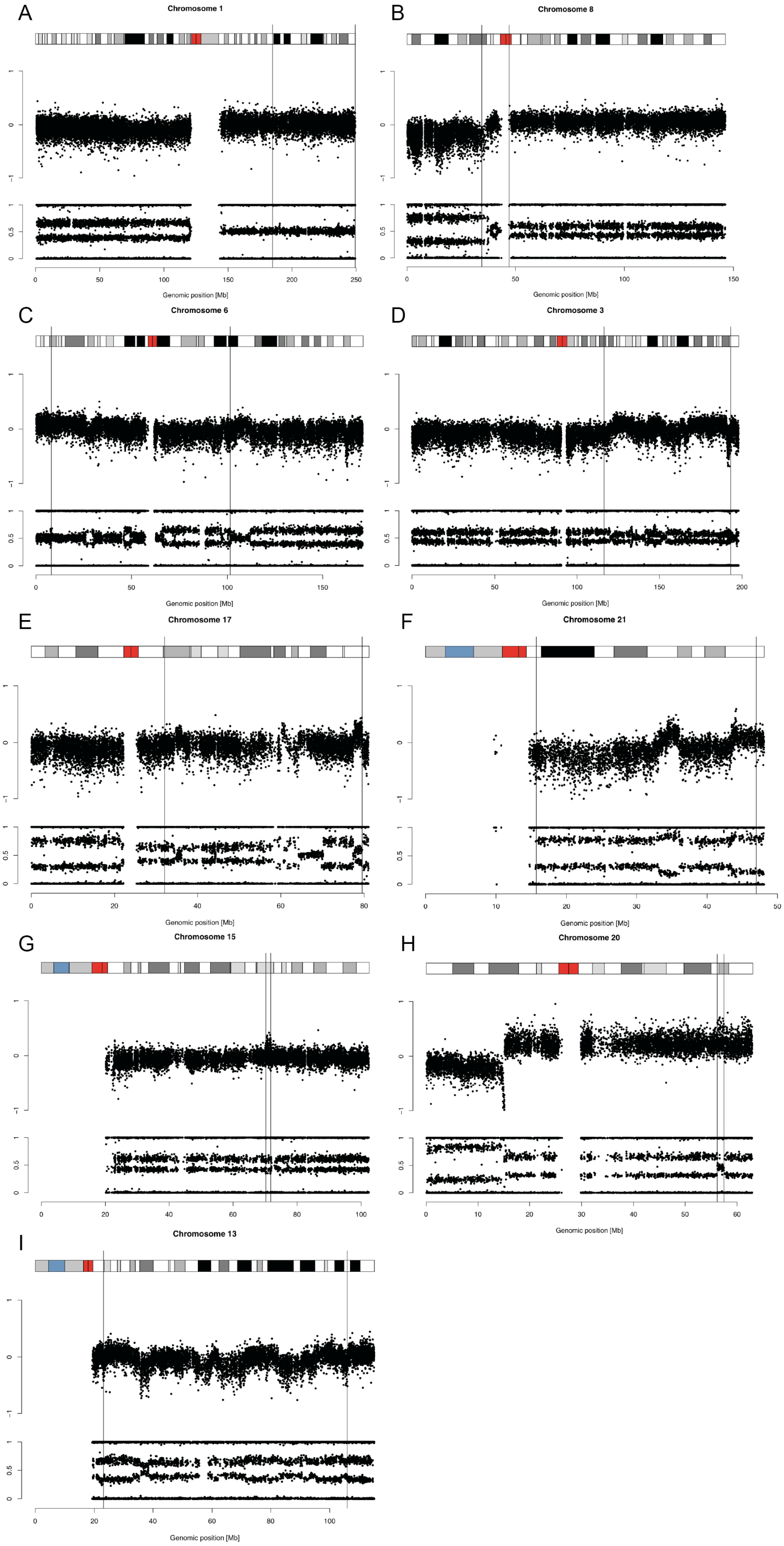

Log R ratios and B allele frequencies for chromosomes affected by chromothripsis. (A) Chr1 cluster, patient 3, metastasis. (B) Chr8 cluster, patient 4, primary tumor. (C) and (D) Chr3-6 cluster, patient 4, primary tumor. (E) and (F) Chr17-21 cluster, patient 4, metastasis. (G) and (H) Chr15-20 cluster, patient 3, primary tumor. (I) Chr13 cluster, patient 1, primary tumor. Vertical lines indicate the boundaries of complex clusters. Chromosome ideograms are depicted in the horizontal bar at the top of each panel.
