# Supplementary material for: Chromothripsis is a common mechanism driving genomic rearrangements in primary and metastatic colorectal cancer
Source: Genome Biol. 2011 Oct 19;12(10):R103. doi: 10.1186/gb-2011-12-10-r103 (PMC3333773; doi:10.1186/gb-2011-12-10-r103)
Supplement: Additional file 9 — PCR gel of genomic rearrangements within clusters on chromosomes 17 and 21 and chromosomes 3 and 6. [file gb-2011-12-10-r103-S9.PDF]

Additional data file 9

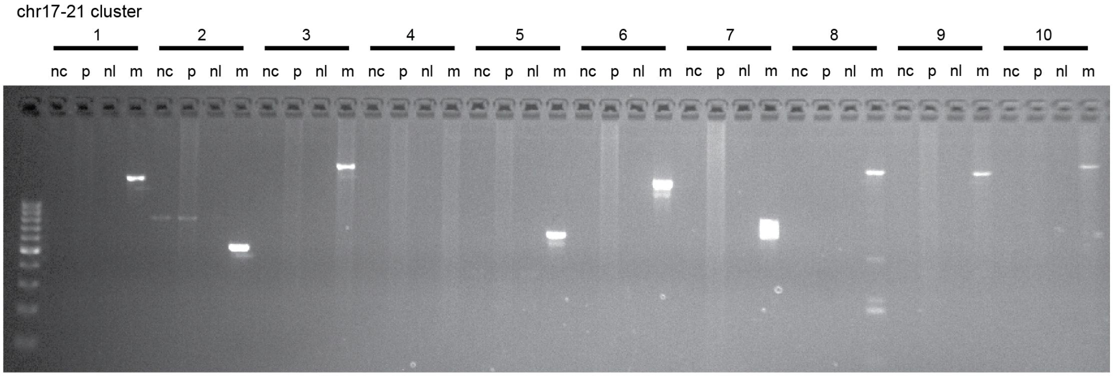

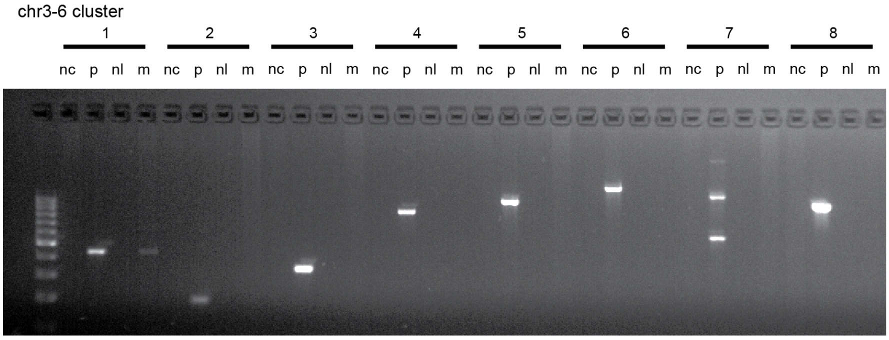

PCR gel of genomic rearrangements within clusters on chromomomes 17 and 21 (upper panel) and chromosome 3 and 6 (lower panel). nc, normal colon tissue; p, primary colon tumor; nl, normal liver tissue; m, liver metastasis. We analyzed the lesion-specific presence of 222 rearrangements by PCR across the rearrangement breakpoint.
