# Supplementary material for: Chromothripsis is a common mechanism driving genomic rearrangements in primary and metastatic colorectal cancer
Source: Genome Biol. 2011 Oct 19;12(10):R103. doi: 10.1186/gb-2011-12-10-r103 (PMC3333773; doi:10.1186/gb-2011-12-10-r103)
Supplement: Additional file 10 — Table indicating the presence of complex rearrangement clusters in primary and metastatic tumors. [file gb-2011-12-10-r103-S10.PDF]

Additional data file 10

Presence of complex rearrangement clusters in primary and metastatic tumor samples

cluster	patient	presence
chr3_chr6	patient 4	primary tumor
chr1	patient 3	metastasis
chr17_chr21	patient 4	metastasis
chr8	patient 4	primary tumor and metastasis (partially)
chr13	patient 1	primary tumor and metastasis
chr15_chr20	patient 3	primary tumor
