# Supplementary material for: Chromothripsis is a common mechanism driving genomic rearrangements in primary and metastatic colorectal cancer
Source: Genome Biol. 2011 Oct 19;12(10):R103. doi: 10.1186/gb-2011-12-10-r103 (PMC3333773; doi:10.1186/gb-2011-12-10-r103)
Supplement: Additional file 11 — Sequence characteristics of tumor-specific fusion points. [file gb-2011-12-10-r103-S11.PDF]

Additional data file 11

A

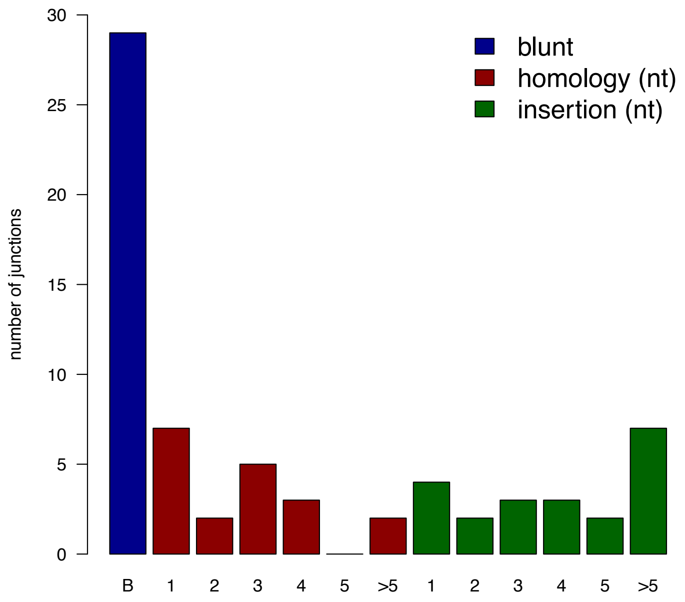

B

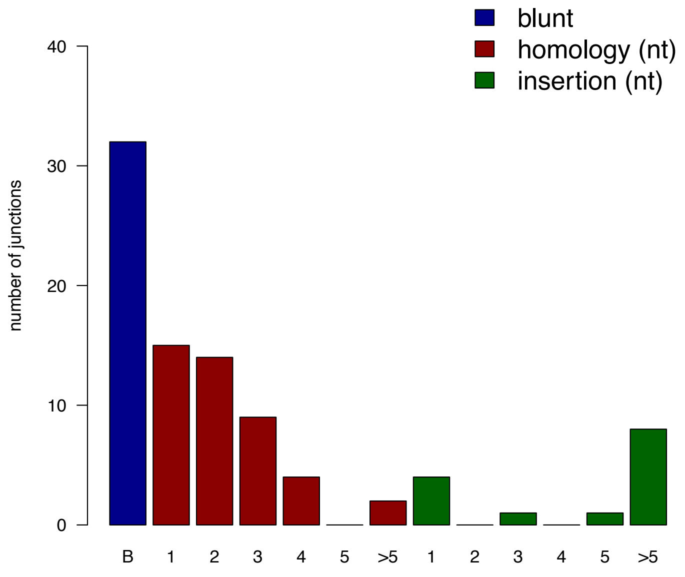

Sequence characteristics for 159 tumor-specific fusion points. (A) Characteristics of fusion points involving chromothripsis clusters (n=69). (B) Characteristics of fusion points outside of chromothripsis clusters (n=90). The distribution across different types of characteristics is similar for fusion points within or outside chromothripsis clusters. For the majority of fusion points we see no or very little microhomology or inserted sequences, which suggests that non-homologous end-joining accounted for the repair process.
