# Supplementary material for: Chromothripsis is a common mechanism driving genomic rearrangements in primary and metastatic colorectal cancer
Source: Genome Biol. 2011 Oct 19;12(10):R103. doi: 10.1186/gb-2011-12-10-r103 (PMC3333773; doi:10.1186/gb-2011-12-10-r103)
Supplement: Additional file 12 — Hotspots of rearrangements in PARK2 and MACROD2. [file gb-2011-12-10-r103-S12.PDF]

Additional data file 12

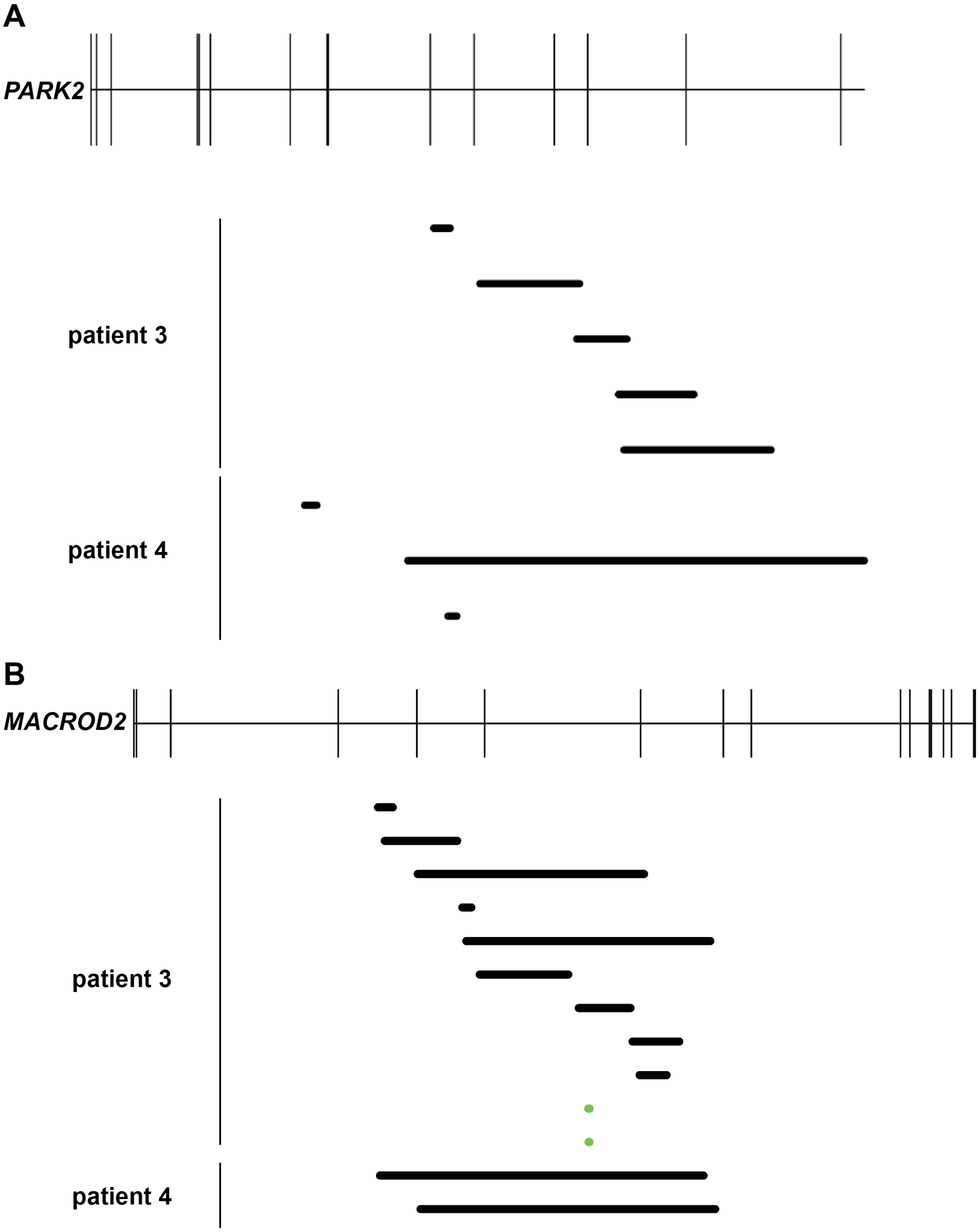

Hotspots of rearrangements in *PARK2* and *MACROD2*. (A) Recurrent deletions in *PARK2* in patients 3 and 4. (B) Recurrent deletions and rearrangement breakpoints in *MACROD2* in patient 3 and 4. Green spots indicate rearrangement breakpoints and black lines indicate deletions. Exons are depicted as vertical bars.
